# Editorial

**Published:** 2015-09-01

**Authors:** A. R. Mehdizadeh, S. M. J. Mortazavi

**Affiliations:** 1 Deputy Editor in Chief, Department of Medical Physics, School of Medicine, Shiraz University of Medical Science, Shiraz, Iran; 2 Medical Physics and Medical Engineering Department, School of Medicine, Shiraz University of Medical Sciences, Shiraz, Iran

Today, due to the explosion of electricity-based technological applications, the use of electricity at home and at work has become an inseparable part of everyday life. Electricity flow generates electric and magnetic fields. Since the late 1970s there is a growing concern about the health effects of exposure to these electric and magnetic fields. As World Health Organization (WHO) states, although exposure to electromagnetic fields (EMFs) is not a new phenomenon, over the past decades due to growing electricity demand, environmental exposure to man-made EMFs has increased rapidly. On the other hand, ever-advancing technologies and cardinal changes in social behavior have created a wide variety of artificial sources of EMFs ranging from telecommunications and broadcasting to domestic appliances and industrial equipment. In 1996, WHO, in response to growing public health concerns over possible health effects of the exposure to different EMF sources, launched the international EMF project as a large, multidisciplinary research program. Despite controversies, the effects of EMF exposure on general health, pregnancy outcome, cataract and cancer are well documented. On the other hand, as there are people who report to be “hypersensitive” to EMFs, research on this topic is continuing around the world. Over the past several years, our labs at the Ionizing and Non-ionizing Radiation Protection Research Center (INIRPRC), Shiraz University of Medical Sciences (SUMS) have focused on studying the health effects of exposure of laboratory animals and human to some common sources of electromagnetic fields such as mobile phones [1-7] and their base stations [8], laptop computers [9], MRI [10] and mobile phone jammers [11] as well as occupational exposure to electromagnetic fields generated by cavitrons [5] or Helmholtz coils [12]. 

## SUMS EMF Studies: Seeking Balanced Judgment

Although exposure to EMFs has caused a great deal of concern globally, radiofrequency radiation has many critical applications in both telecommunication and non-communication (including biomedical) fields. No doubt, there are known detrimental effects associated with exposure to radiofrequency radiation (RFF) radiation. However, in some special circumstances (short term- low level exposures), these radiations may cause some potential beneficial effects. Substantial evidence now indicates that the dose response for the biological effects of radiofrequency radiation is possibly non-linear. We know well that low doses of ionizing radiation may induce some beneficial effects such as stimulating the activation of special repair mechanisms. In a similar pattern, other researchers and our team have shown that low levels of RFR may induce some beneficial/stimulatory effects.

Regarding detrimental effects of EMFs, numerous studies showed that exposure to common sources of EMF such as mobile phones [13-15], mobile phone jammers [11], laptops [9] or wireless internet-connected laptops [17] or extremely low frequency electromagnetic field (ELFs) [18] decreased human sperm quality. In spite of the fact that there are known detrimental effects associated with exposure to RFR, in some special circumstances (short term- low level exposures), these radiations may cause some potential beneficial effects other than the induction of adaptive response [19]. We have shown that low levels of RFR as a non-ionizing radiation, can also induce stimulatory/beneficial effects. It was reported by our team that the visual reaction time of university students significantly decreased after a 10 min exposure to radiofrequency radiation emitted by a mobile phone [20]. It was also previously shown that short-term exposure of elementary school students to RFR radiation might led to the better performance of their short-term memory [21]. On the other hand, it has also been shown that occupational exposures to radar radiations decrease the reaction time in radar workers [22]. 

Furthermore, some recent reports have indicated that RFR radiation may have a role in protecting against cognitive impairment in Alzheimer’s disease [23, 24]. Furthermore, it has been shown that pre-exposure of mice to radiofrequency radiation emitted by a GSM mobile phone increased their resistance to a subsequent Escherichia coli infection [25, 26]. This phenomenon may have very important applications in long term space missions. On the other hand, we showed that exposure to microwave radiation may induce a significant survival adaptive response after exposure to lethal doses of gamma rays [27]. In this issue of the Journal of JBPE, we focus on the health effects of exposure to different sources of electromagnetic fields. We would like to sincerely thank Mrs. Rajabpour for her tireless efforts and valuable assistance in preparing this issue in a timely fashion. 
